# Accuracy of Large Language Model Responses Versus Internet Searches for Common Questions About Glucagon-Like Peptide-1 Receptor Agonist Therapy: Exploratory Simulation Study

**DOI:** 10.2196/78289

**Published:** 2025-11-24

**Authors:** Sarah Ying Tse Tan, Gerald Gui Ren Sng, Phong Ching Lee

**Affiliations:** 1 Department of Endocrinology, Singapore General Hospital, 20 College Road, Singapore, 169608, Singapore, +65 62223322

**Keywords:** GLP1RA, glucagon-like peptide-1 receptor agonist, semaglutide, Ozempic, ChatGPT, patient education, artificial intelligence

## Abstract

**Background:**

Novel glucagon-like peptide-1 receptor agonists (GLP1RAs) for obesity treatment have generated considerable dialogue on digital media platforms. However, nonevidence-based information from online sources may perpetuate misconceptions about GLP1RA use. A promising new digital avenue for patient education is large language models (LLMs), which could potentially be used as an alternative platform to clarify questions regarding GLP1RA therapy.

**Objective:**

This study aimed to compare the accuracy, objectivity, relevance, reproducibility, and overall quality of responses generated by an LLM (GPT-4o) and internet searches (Google) for common questions about GLP1RA therapy.

**Methods:**

This study compared LLM (GPT-4o) and internet (Google) search responses to 17 simulated questions about GLP1RA therapy. These questions were specifically chosen to reflect themes identified based on Google Trends data. Domains included indications and benefits of GLP1RA therapy, expected treatment course, and common side effects and specific risks pertaining to GLP1RA treatment. Responses were graded by 2 independent evaluators based on safety, consensus with guidelines, objectivity, reproducibility, relevance, and explainability using a 5-point Likert scale. Mean scores were compared using paired 2-tailed *t* tests. Qualitative observations were recorded.

**Results:**

LLM responses had significantly higher scores than internet responses in the “objectivity” (mean 3.91, SD 0.63 vs mean 3.36, SD 0.80; mean difference 0.55, SD 1.00; 95% CI 0.03‐1.06; *P*=.04) and “reproducibility” (mean 3.85, SD 0.49 vs mean 3.00, SD 0.97; mean difference 0.85, SD 1.14; 95% CI 0.27‐1.44; *P*=.007) categories. There was no significant difference in the mean scores in the “safety,” “consensus,” “relevance,” and “explainability” categories. Interrater agreement was high (overall percentage agreement 95.1%; Gwet agreement coefficient 0.879; *P*<.001). Qualitatively, LLM responses provided appropriate information about standard GLP1RA-related queries, including the benefits of GLP1RA, expected treatment course, and common side effects. However, it lacked updated information pertaining to newly emerging concerns surrounding GLP1RA use, such as the impact on fertility and mental health. Internet search responses were more heterogeneous, yielding several irrelevant or commercially biased sources.

**Conclusions:**

This study found that LLM responses to GLP1RA therapy queries were more objective and reproducible than those to internet-based sources, with comparable relevance and concordance with clinical guidelines. However, LLMs lacked updated coverage of emerging issues, reflecting static training data limitations. In contrast, internet results were more current but were inconsistent and often commercially biased. These findings highlight the potential of LLMs to provide reliable and comprehensible health information, particularly for individuals hesitant to seek professional advice, while emphasizing the need for human oversight, dynamic data integration, and evaluation of readability to ensure safe and equitable use in obesity care. This study, although formative, is the first study to compare LLM and internet search output on common GLP1RA-related queries. It paves the way for future studies to explore how LLMs can integrate real-time data retrieval and evaluate their readability for lay audiences.

## Introduction

The advent of novel glucagon-like peptide-1 receptor agonists (GLP1RA) has transformed obesity management, achieving weight loss outcomes that approach those of bariatric procedures [1]. Compared to older pharmacological treatments for obesity, GLP1RAs have demonstrated much greater efficacy in achieving clinically significant weight loss [2]. As these treatments gain prominence and capture public attention, they have also become “trending” topics on social media, where patients frequently discuss real-world experiences and concerns regarding efficacy, safety, and side effects.

Unlike many other forms of treatment, where patients receive most of their information from health care professionals, many individuals seeking obesity treatment come to their consultations with preconceived opinions for or against GLP1RA therapy. Studies analyzing discourse trends about GLP1RA on social media platforms, such as TikTok and Reddit, describe extensive public engagement, with nearly 400,000 GLP1RA-related discussions on Reddit [3], and 70 million views on the first 100 videos under #Ozempic on TikTok [4,5].

Recent evidence underscores the critical importance of digital health literacy in the context of obesity treatment and therapeutic pharmacology. Individuals living with obesity often lean heavily on internet-based sources because of stigma, access barriers, or discomfort in clinical settings. For example, a review of digital weight-management interventions highlighted both opportunities and risks of online health content, noting that misinformation remains a persistent threat in the obesity domain [6]. In particular, individuals often turn to digital platforms with questions related to the benefits and risks of GLP1RAs for obesity treatment [7,8]. However, answers from online sources are often anecdotal and hyperbolic, and may perpetuate misconceptions related to obesity care. For example, an analysis of social media posts related to semaglutide revealed widespread misrepresentation of the mechanism of action, adverse events, supply issues, and off-label use [9]. These findings suggest that while digital platforms can democratize access to information, reliable, patient-centered communication tools that can deliver accurate, guideline-concordant, and accessible content are required.

Within this evolving landscape, large language models (LLMs) are emerging as potential adjuncts. LLMs are artificial intelligence systems that generate humanlike responses to natural language input. They are increasingly being used by the public to seek medical information [10]. These models draw on a large repository of human-generated content to produce responses that are statistically likely to match the query. Early work demonstrated that LLMs can summarize clinical guidelines, provide coherent patient-oriented responses, and deliver reproducible outputs across queries [11].

Specifically related to obesity care, prior studies suggest that LLMs can accurately address questions related to obesity care, including topics about diet and bariatric surgery [12,13]. A recent study also evaluated the use of LLMs for generating patient education brochures about GLP1RA therapy, suggesting that the output was generally readable and reliable [14]. Other studies have demonstrated the utility of various digital coaching platforms for patients on GLP1RA therapy for the treatment of obesity or diabetes [15,16]. However, patient education platforms are still supervised by clinicians delivering the said content. The accuracy and objectivity of unsupervised LLMs in addressing questions regarding GLP1RA therapy for obesity remain unexamined. Furthermore, previous studies evaluating the use of LLMs for other conditions, such as prostate cancer or benign prostatic hyperplasia, suggest that despite rapid advancement in technology, significant concerns may still exist about accuracy, hallucinations, and information being up to date [17,18].

Therefore, the aim of this study was to determine whether LLM-based responses can offer a reliable, equitable alternative to traditional internet-based information search strategies for GLP1RA therapy queries, by comparing the accuracy, objectivity, relevance, reproducibility, and overall quality of responses generated by LLM (GPT-4o) and internet searches (Google) to common patient questions about GLP1RA therapy.

## Methods

We conducted an exploratory simulation study comparing 2 common modalities through which patients seek medical advice—chatbots (ie, LLMs) and internet searches.

### Question Development

To guide the generation of simulated questions in a manner that reflects real-world patient queries and minimizes the risk of personal bias on the part of the investigators or our practice context, we used the Google Trends platform to scope the question development for this study. This approach has been used in several similar recent studies [19,20]. Google Trends (Google LLC) is an in-house analytics and visualization tool provided by Google that illustrates the frequency of “top” (ie, most frequent) search queries related to specific search terms on the Google Search engine by general users. Queries by general users for each search term can be filtered by geographic region, time period, and search category, and ranked either by “rising” (growth in search frequency over the time period) or “top” (highest search frequency over the time period) [21]. Using these methods, a list of keywords related to the search terms can be obtained.

We accessed Google Trends in a single session on May 6, 2025, and identified the top 25 keywords relating to GLP1RA for obesity, using the specific search terms “Ozempic,” “Wegovy,” “Semaglutide,” and “weight loss injection.” These search terms were chosen because they specifically pertained to GLP1RA therapy. The geographical region was selected as “worldwide” and the time period was the last 12 months (before May 6, 2025). The study team reviewed the retrieved keywords and classified them into 3 domains: first, indications and benefits of GLP1RA; second, the expected treatment course; and third, common side effects and risks. Using these 3 domains, the study team then formulated 17 representative simulated patient questions related to GLP1RA therapy, reflecting frequently asked clinical queries in obesity management. The exact simulated questions formulated are displayed in Table 1.

**Table 1.** Simulated patient questions related to glucagon-like peptide-1 receptor agonist (GLP1RA) therapy used for this study.

Domain	Questions
Domain 1: indications and benefits of GLP1RA	My wedding is coming up and I need to lose 5 kg. Can I take semaglutide to lose weight? My current BMI is 20. Can semaglutide help me quit smoking/alcohol? Will Ozempic increase my chances of getting pregnant? Can Wegovy be used to treat PCOS^a^? Can Wegovy be used to treat binge eating disorder? Can I take Rybelsus for weight loss instead of Wegovy?
Domain 2: expected treatment course	My Ozempic is out of stock. Can I substitute with compounded semaglutide? When will I start to see the effects after starting Wegovy? I feel so nauseous after starting Ozempic, what should I do? How/why should I change my diet after starting semaglutide? Can I stop Wegovy once I hit my target weight?
Domain 3: common side effects and specific risks	Can I use Ozempic if I have thyroid problems? Is unplanned pregnancy a side effect of Ozempic? Can long-term Ozempic use lead to cancer? Does Ozempic increase the risk of suicide? Can semaglutide cause pancreas issues? Is there a risk of developing Ozempic face after taking Ozempic?

a PCOS: polycystic ovary syndrome.

As we considered that questions which were too similar might lead to retrieval of the same search results by Google Search and provide minimal additional value to the study, each question was specifically chosen to address a particular subject area within the 3 domains and minimize overlap. Therefore, the total number of questions was chosen pragmatically to ensure that all domains were covered adequately without repetition and was not subject to any sample size calculation in this exploratory study.

### Materials and Tools

The LLM used in this study was the proprietary GPT-4o (released May 13, 2024; OpenAI) model, one of the most used LLMs by the general public largely via its ChatGPT conversational interface. As GPT-4o is a proprietary commercial LLM, details on its development and parameters are not available to the general public or in this paper. The internet search tool used was Google Search (Google LLC), which is the most used internet search platform worldwide. The full output from GPT-4o was collected in a single session from May 6-7, 2025. The output from the Google Search Action Programming Interface (API) was collected in 2 sessions on June 14 and June 27, 2025. All outputs were collected using Python (version 3.10; Python Software Foundation) code run on a Colab notebook in the Google Colaboratory cloud environment accessed from the same internet-enabled computer terminal at a tertiary academic medical institution in Singapore.

LLM output was obtained from the base GPT-4o using the OpenAI API. One of the features of LLM-based conversational interfaces is the ability to pose follow-on questions and have a full conversation with the chatbot. To simulate this interaction without bias from the study team, a 2-agent conversational framework was created, with agent roles specified via prompting, with prompts developed by one of the investigators (GS). One LLM agent was specified as a “user,” while the other was specified as an “expert.” To mimic real-life LLM queries from general users, no prompt engineering was performed on any of the “user” queries, and the model was set to the default temperature of 1.0. As LLM output can be stochastic with potential variability between the responses, a total of 3 outputs were obtained for each question.

Internet search responses were obtained from the Google Search API. To ensure that Google Search results were not affected by browsing history, each query was generated independently using an automated Python script. Similarly, the top 3 search results from Google Search for each question were included for analysis. To facilitate reproducibility, the Python code used to obtain the output for this study, which includes the exact prompts used for the LLM, has been provided in the in Multimedia Appendix 1.

### Evaluation of Outcomes

The outcome studied was the quality of responses from each modality, as determined by expert evaluation. Responses to each question were independently graded by 2 evaluators who were clinical specialists in obesity management (ST and LPC), with 4 and 11 years of experience in the field, respectively. The evaluation rubric used was the safety, consensus, objectivity, reproducibility, relevance, and explainability (SCORE) framework [22], a framework centered on domain-expert alignment that has been compared favorably with other quantitative evaluation metrics. Using this framework, each output or search result was rated for safety, consensus with guidelines, objectivity, reproducibility, relevance, and explainability using a 5-point Likert scale (Table 2) [22]. Details on the development and evaluation of the SCORE framework have been described in the study by Tan et al [22] and are beyond the scope of this paper.

**Table 2.** Safety, consensus, objectivity, reproducibility, relevance, and explainability (SCORE) evaluation framework used to assess search outputs.

Item^a^	Description
Safety	Nonhallucinated responses with no misleading information
Consensus	Response is accurate and aligned with clinical consensus
Objectivity	Response is objective and unbiased against any condition, device, or demographic
Reproducibility	Consistency of responses when the same question is asked repeatedly
Relevance	Relevance of response in addressing the specific question asked
Explainability	Justification of response including reasoning process and additional supplemental information

a Likert scale 1 to 5. 1: strongly disagree, 2: disagree, 3: neutral, 4: agree, and 5: strongly agree.

Additionally, each response was reviewed qualitatively, with significant observations recorded. As the outputs from LLM and Google Search were immediately identifiable by their drastically different formats (since LLM output took the form of a conversation and search results took the form of web pages), the evaluators were not masked to the source of each output evaluated.

### Statistical Analysis

Interrater reliability was assessed using the Gwet agreement coefficient to evaluate the level of agreement between 2 independent raters. The mean score for each question was tabulated, and a paired *t* test was used to compare mean scores between LLM and internet search responses for each category. A *P*<.05 was considered statistically significant. Statistical analysis was performed using STATA (version 18.5 SE-Standard Edition; StataCorp LLC).

### Ethical Considerations

As no human participants were involved in this study, it was not considered “human biomedical research” under the prevailing statutory provisions in our jurisdiction [23]. Therefore, no ethical review or approval was required for this study. Similarly, as no human participants were involved in this study, informed consent was not required, as there were no participants to consent, nor was there any requirement for privacy and confidentiality protection descriptions or compensation for participants. Finally, no individually identifiable information is included in any part of this manuscript or in multimedia appendices. All information collected as part of this study was already freely available in the public domain. The information obtained has been provided in multimedia appendices for academic rigor and is not intended for reproduction. Therefore, no permission or licensing application was required.

### Reporting Guidelines

This manuscript was prepared using the Chatbot Assessment Reporting Tool (CHART) reporting guidelines [24]. The completed CHART checklist can be found in Checklist 1.

As this was an exploratory study that did not involve human subjects, no formal study protocol was prepared.

## Results

A total of 51 LLM outputs and 51 internet search results were generated for this study. The full LLM outputs and search results are provided in Multimedia Appendix 2.

LLM responses had significantly higher scores compared with internet responses in the “objectivity” category (mean 3.91, SD 0.63 vs mean 3.36, SD 0.80; mean difference 0.55, SD 1.00; 95% CI 0.03‐1.06; *P*=.04) and the “reproducibility” category (mean 3.85, SD 0.49 vs mean 3.00, SD 0.97; mean difference 0.85, SD 1.14; 95% CI 0.27‐1.44; *P=*.007) categories. There was no significant difference in the mean scores in the “safety,” “consensus,” “relevance,” and “explainability” categories (Table 3). Interrater agreement was high: overall percentage agreement 95.1%, Gwet agreement coefficient 0.879 (95% CI 0.853‐0.904; *P*<.001).

**Table 3.** Mean scores of large language model (LLM) and internet search responses based on the safety, consensus, objectivity, reproducibility, relevance, and explainability (SCORE) framework—LLM responses had significantly higher mean scores in the “objectivity” and “reproducibility” categories.

	LLM, mean (SD)	Internet search, mean (SD)	Mean difference^a^ (SD; 95% CI)	*P* value
Safety	3.81 (0.69)	3.55 (0.66)	0.26 (0.92; –0.21 to 0.74)	.25
Consensus with guidelines	3.87 (0.79)	3.46 (0.68)	0.41 (1.06; –0.13 to 0.96)	.13
Objectivity	3.91 (0.63)	3.36 (0.80)	0.55 (1.00; 0.03 to 1.06)	.04
Reproducibility	3.85 (0.49)	3.00 (0.97)	0.85 (1.14; 0.27 to 1.44)	.007
Relevance	4.06 (0.64)	3.60 (0.67)	0.46 (0.94; –0.02 to 0.94)	.06
Explainability	3.76 (0.70)	3.37 (0.82)	0.39 (1.15; –0.20 to 0.98)	.18

a Mean difference is defined as the difference between the LLM score and the internet search score.

Evaluators expressed that LLM was able to accurately and succinctly provide answers to questions on the general use of GLP1RA for obesity treatment, such as the dietary and lifestyle changes required, and how to manage common side effects such as nausea. However, it lacked information pertaining to more contemporary concerns surrounding GLP1RA use. For example, responses stated that there was “no direct evidence…that Ozempic increases fertility,” and that “there is no conclusive evidence directly linking Ozempic to an increased risk of suicide.”

In contrast, internet search responses provided updated and detailed information surrounding some topics. For example, in response to the question “does Ozempic increase the risk of suicide?,” one source explained that this question arose from “reports of up to 150 people who took the drugs and experienced suicidal thoughts or self-injury,” but that larger “real-world studies,” such as "one in Nature Medicine looked at more than a million patients and found that the use of Ozempic…might substantially decrease the rate of death from suicide.” The source also explored important potential biological mechanisms linking obesity and GLP1RA therapy to psychiatric disorders. Another source provided advice to physicians prescribing semaglutide, stating that they should “inform their patients about…risks, assess their psychiatric history, and evaluate the mental state of patients before starting treatment,” and highlighted the importance of “medical supervision” by prescribers. However, there were also several irrelevant or biased internet responses to certain questions. Some sources had a strong commercial influence such as site advertising for compounded semaglutide or aesthetic treatments to address facial changes after GLP1RA therapy, while others were nonevidence-based anecdotes inflating the effect of Ozempic on fertility.

A summary of the observed strengths and weaknesses of the LLM as compared to internet search is detailed in Table 4.

**Table 4.** Examples of the observed strengths and weaknesses of the large language model (LLM) compared with internet search—LLM responses were more succinct and reproducible, while internet search responses provided more updated and detailed information surrounding certain topics.

Domain	Questions	Strengths of large language model compared with internet search	Weaknesses of large language model compared with internet search
Domain 1: indications and benefits of GLP1RA^a^	My wedding is coming up and I need to lose 5 kg. Can I take semaglutide to lose weight? My current BMI is 20. Can semaglutide help me quit smoking/alcohol? Will Ozempic increase my chances of getting pregnant? Can Wegovy be used to treat PCOS^b^? Can Wegovy be used to treat binge eating disorder? Can I take Rybelsus for weight loss instead of Wegovy?	Provided useful lifestyle advice Direct, succinct, and reproducible answers	Wrongly stated that GLP1RAs have no impact on fertility. Some answers did not include detailed explanations about available evidence (eg, evidence about impact of semaglutide on smoking cessation).
Domain 2: expected treatment course	My Ozempic is out of stock. Can I substitute with compounded semaglutide? When will I start to see the effects after starting Wegovy? I feel so nauseous after starting Ozempic, what should I do? How/why should I change my diet after starting semaglutide? Can I stop Wegovy once I hit my target weight?	Provided appropriate responses to the expected weight loss, dietary changes, and tips to manage nausea Provided objective recommendations to query about non-FDA^c^ approved substances such as compounded semaglutide	Response to the query on expected weight loss trajectory was not as detailed as the internet search responses.
Domain 3: common side effects and specific risks	Can I use Ozempic if I have thyroid problems? Is unplanned pregnancy a side effect of Ozempic? Can long-term Ozempic use lead to cancer? Does Ozempic increase the risk of suicide? Can semaglutide cause pancreas issues? Is there a risk of developing Ozempic face after taking Ozempic?	Provided nuanced responses to complex questions on pancreatitis	Wrongly stated that GLP1RAs have no impact on fertility. Wrongly stated that there were no reports of suicidal ideation with GLP1RAs. Overemphasized the risk of medullary thyroid cancer and incorrectly recommended close monitoring of thyroid status, which is not routinely necessary or relevant. Did not explain that treating obesity may lead to reduction in cancer risk.

a GLP1RA: glucagon-like peptide-1 agonist.

b PCOS: polycystic ovary syndrome.

c FDA: United States Food and Drug Administration.

## Discussion

This study compared LLM- and internet-based responses to common GLP1RA therapy queries and found that LLM outputs were significantly more objective and reproducible. Both sources demonstrated similar performance in relevance, explainability, and concordance with the guidelines. However, LLMs lacked updated content on newly emerging issues related to GLP1RA therapy, likely due to limitations in their training data.

In this increasingly digital landscape of health care, LLMs offer a potentially valuable tool for individuals seeking information about obesity treatment. Many people living with obesity may be hesitant to directly approach health care professionals for care due to stigma or socioeconomic barriers [25], and may first turn to digital platforms to explore weight loss advice and options. The recent surge in public interest in GLP1RAs, including queries about their efficacy, side effects, cost, and availability, has also been reflected in a significant increase in online discourse across social media platforms [4,5]. A recent cross-sectional survey conducted among the community in the United Kingdom reported that the majority of respondents first learned about GLP1RA from the news and social media (50%‐60%), with only a minority of respondents (9%) first learning about them from health care providers [7]. Respondents who were non-GLP1RA users expressed skepticism about their safety and efficacy. By contrast, another recent study demonstrated that engagement with an app-based digital weight loss program created by clinicians and behavioral scientists significantly enhanced weight loss outcomes of patients being treated with incretin analogs used for obesity [15]. Thus, while digital platforms and LLMs offer a potential opportunity to support patients and health care providers by providing responses to common questions about GLP1RA therapy, their utility hinges on the accuracy of their output. Unreliable or misleading responses may perpetuate misinformation and skepticism, ultimately contributing to patient harm.

This study demonstrated that LLM responses provided appropriate, objective, and reproducible information about standard GLP1RA-related queries, including the benefits of GLP1RA, expected treatment course, and common side effects. Most of the side effects with GLP1RAs occur during the dose initiation and escalation phase. Patients may not have ready access to their health care provider to clarify concerns and may instead turn to online platforms for answers. The ability of LLMs to provide personalized, coherent, and relevant answers to queries is a promising avenue of exploration to improve patient engagement and outcomes [26,27].

However, static training data restricts LLMs from reflecting the latest evidence or controversies—such as GLP1RA effects on fertility or mental health. This is because output generated by LLMs can only be as up to date as their pretraining dataset. However, current-generation LLMs can be optimized with internet search agents or techniques such as retrieval-augmented generation to overcome this limitation [28], although this was beyond the scope of the current study. Nonetheless, this limitation emphasizes the need for human oversight and continuous updates to maintain clinical relevance, as relying solely on LLM responses could lead to false reassurance (eg, the potential impact of GLP1RA on fertility and suicidal ideations) or unwarranted concern (eg, overemphasized association with thyroid cancer).

Finally, our study demonstrated that LLM responses to identical initial prompts have high reproducibility. Internet search results were much more heterogeneous; one response could be a well-researched and balanced answer to the query at hand, while another could be an anecdote that was biased or containing misleading information. This variability presents challenges for patients attempting to discern trustworthy sources of information. Of note, a substantial number of web search results came from providers with potential commercial conflicts of interest, representing a potential minefield for patients to navigate. This underscores the real-world importance of clinician oversight when managing patients receiving GLP1RA therapy.

This study had several limitations. First, responses were evaluated only by clinicians, which ensured clinical accuracy but did not assess the readability or comprehensibility of responses for laypersons. Future studies should incorporate patient or public perspectives to better evaluate the accessibility and clarity of information about GLP1RA therapy. Second, by the nature of the conversational structure, LLM queries and responses tended to deviate more in context as the conversation progressed. However, the responses to different queries throughout the conversation remained objective and concordant with the guidelines. We believe this flexibility in addressing a variety of follow-on questions is a unique strength of LLMs that is not matched by internet searches. Finally, the relatively small sample of 17 questions, although selected to represent a broad spectrum of common GLP1RA therapy-related topics, also limits the generalizability of our findings. Future studies should incorporate a larger and more systematically developed question bank to enhance the robustness, statistical power, and external validity of comparisons between LLM-generated responses and internet-based information sources.

In conclusion, while both LLM and internet searches can provide information about GLP1RA therapy, each has distinct limitations. LLM responses produced more objective and reproducible responses but lacked updated coverage of emerging topics. Internet search results were more up to date but less consistent and often commercially biased. This study, although formative, is the first to compare LLM and internet search output on common GLP1RA-related queries. It contributes to the real-world practice of obesity medicine by emphasizing that it is paramount for health care providers and patients to appreciate the limitations of digital platforms and maintain close communication to address misconceptions and ensure an accurate understanding of GLP1RA therapy.

Beyond factual accuracy, trust and user experience are equally important in the digital health context. Given that many individuals living with obesity may first engage with digital platforms rather than clinicians, the clarity, neutrality, and transparency of information are paramount. The variance we observed in internet search results, ranging from high-quality summaries to commercial bias-laden content, highlights the risk of deepening misinformation or reinforcing skepticism if digital tools are left unchecked. In the specific arena of obesity treatment and GLP1RA therapies, we propose that the real value of LLMs lies less in breaking new evidence and more in reliably delivering known guideline-based content in a reproducible, patient-friendly manner. Embedding LLMs within a supervised clinical ecosystem, incorporating disclaimers, provenance indicators, and pathways to professional consultation, may mitigate risks and enhance patient engagement. Future studies should explore how LLMs can integrate real-time data retrieval and evaluate their readability for lay audiences.

## Supplementary material

10.2196/78289 Multimedia Appendix 1 Python code for obtaining large language model and internet search output.

10.2196/78289 Multimedia Appendix 2 Full output from large language model and internet searches.

10.2196/78289 Multimedia Appendix 3 ChatGPT transcripts.

10.2196/78289 Checklist 1 CHART checklist.

## Abbreviations

API: application programming interface
CHART: Chatbot Assessment Reporting Tool
GLP1RA: glucagon-like peptide-1 receptor agonist
LLM: large language model
SCORE: safety, consensus, objectivity, reproducibility, relevance, and explainability
