# Supplementary material for: Accuracy of Large Language Model Responses Versus Internet Searches for Common Questions About Glucagon-Like Peptide-1 Receptor Agonist Therapy: Exploratory Simulation Study
Source: JMIR Form Res. 2025 Nov 24;9:e78289. doi: 10.2196/78289 (PMC12643393; doi:10.2196/78289)
Supplement: Multimedia Appendix 1 [file formative-v9-e78289-s001.docx]

**Supplementary Material 3 – Python Code for obtaining LLM and Google Search output**

**LLM Output**

| !pip install openai  import pandas as pd  import os  from datetime import datetime  csv_path = {path}  log_dir = {path}  os.makedirs(log_dir, exist_ok=True)  df = pd.read_csv(csv_path, header=None)  questions = df[0].dropna().tolist()  print("\n Extracted Questions:")  for i, q in enumerate(questions, 1):      print(f"{i}. {q}")  import os  from openai import OpenAI  os.environ["OPENAI_API_KEY"] = {OpenAI key}  USER_AGENT_INSTRUCTION = """  You are a person living with obesity. Your knowledge is limited to general understanding and what the average person would be expected to know.  You will continue to ask follow-up questions to each response until you believe the initial question has been adequately answered.  Each follow-up question should be between one to three sentences long.  """  SYSTEM_ROLE_DESCRIPTION = "””  You are an expert providing clear, helpful, evidence-based answers to questions about obesity and related topics.  "””  client = OpenAI()  def run_conversation(initial_question, max_turns=5, output_file=None):      system_history = [{"role": "system", "content": SYSTEM_ROLE_DESCRIPTION}]      user_history = [{"role": "system", "content": USER_AGENT_INSTRUCTION}]      user_input = initial_question      turn = 0      conversation_log = f"\n=== Starting Conversation: {initial_question} ===\n"      while turn < max_turns:          system_history.append({"role": "user", "content": user_input})          system_response = client.chat.completions.create(              model="gpt-4o",              messages=system_history          ).choices[0].message.content          print(f"\n🧠 System: {system_response}")          conversation_log += f"\n🧠 System: {system_response}\n"          user_history.append({"role": "user", "content": system_response})          follow_up = client.chat.completions.create(              model="gpt-4o",              messages=user_history          ).choices[0].message.content          print(f"\n🙋‍♂️ User: {follow_up}")          conversation_log += f"\n🙋‍♂️ User: {follow_up}\n"          user_input = follow_up          turn += 1      if output_file:          with open(output_file, "w", encoding="utf-8") as f:              f.write(conversation_log + "\n")  for idx, q in enumerate(questions, 1):      output_filename = os.path.join(log_dir, f"Question_{idx}.txt")      run_conversation(q, output_file=output_filename)  print(f"\n All conversations saved to individual files in {log_dir}") |
| --- |

**Google Search output**

| !pip install googlesearch-python beautifulsoup4 pandas  import pandas as pd  csv_path = {path}  questions_df = pd.read_csv(csv_path, header=None)  questions = questions_df[0].tolist()  from googlesearch import search  search_results = {}  for question in questions:      urls = list(search(question, num_results=3))      search_results[question] = urls  from bs4 import BeautifulSoup  import requests  def fetch_page_content(url):      try:          response = requests.get(url, timeout=10)          soup = BeautifulSoup(response.text, 'html.parser')          return soup.get_text(separator=' ', strip=True)      except Exception as e:          return f"Error fetching content: {e}"  content_results = {}  for question, urls in search_results.items():      content_results[question] = {url: fetch_page_content(url) for url in urls}  output_path = {path}  results_df = pd.DataFrame([      {'question': q, 'url': url, 'content': content}      for q, urls in content_results.items()      for url, content in urls.items()  ])  results_df.to_csv(output_path, index=False)  print(f"Results saved to {output_path}") |
| --- |
