# Supplementary material for: Accuracy of Large Language Model Responses Versus Internet Searches for Common Questions About Glucagon-Like Peptide-1 Receptor Agonist Therapy: Exploratory Simulation Study
Source: JMIR Form Res. 2025 Nov 24;9:e78289. doi: 10.2196/78289 (PMC12643393; doi:10.2196/78289)
Supplement: Multimedia Appendix 3 [file formative-v9-e78289-s003.docx]

**Prompt:**

We believe that our manuscript in its current form is sufficiently detailed

However the handling editor insists on lengthening the introduction and discussion sections

Please suggest a few succinct points for each that we can look into and expand for this submission

**Response:**

Here are succinct, high-yield points you can easily expand into additional paragraphs for the Introduction and Discussion—without changing the core structure of your manuscript. These points align with what editors typically look for when they request a “more comprehensive description of the literature,” but can be added concisely without overinflating the paper.

⸻

✅ INTRODUCTION – Add 1–2 Short Paragraphs Based on These Points

1. Growth of GLP-1RA–related misinformation online

• Recent analyses of online discourse (news, TikTok, Instagram, Reddit) show substantial misinformation about GLP-1RA efficacy, risks, and off-label cosmetic use.

• Misinformation volume grew in parallel with rising global prescriptions and supply shortages.

2. Digital health information-seeking patterns

• Surveys demonstrate that most laypeople first learn about GLP-1RAs from online and social media sources, not clinicians.

• People with obesity disproportionately rely on digital platforms due to stigma and healthcare access barriers.

3. Increasing interest in AI-driven health information

• LLMs are increasingly used as first-line sources for health queries.

• Early studies comparing LLM vs web search show LLMs are often more coherent and empathetic but inconsistent in factual accuracy.

4. Gap in literature: No prior study directly comparing LLM vs Internet content for GLP-1RA therapy

• Existing studies focus on:

• Accuracy of LLMs for general medical questions

• Social media misinformation about GLP-1RAs

• Quality of online weight-loss information

• But none examine head-to-head performance on therapeutic queries specific to GLP-1RAs.

⸻

✅ DISCUSSION – Add 1–2 Short Paragraphs Based on These Points

1. Situating findings within broader LLM evaluation research

• Multiple reviews show LLM outputs can be accurate but are limited by training cut-offs and lack of real-time updates.

• Your study aligns with this pattern: strong baseline information, weaker performance on emerging GLP-1RA issues.

2. Implications for patient safety and public health

• Patients encountering inconsistent or commercially biased web content may be misled about:

• Contraindications

• Fertility/mental health concerns

• Expected side-effect timelines

• LLMs may mitigate some of these gaps by offering more consistent content.

3. Importance of integrating retrieval-augmented models

• RAG-enabled LLMs could overcome the static-data limitation.

• Future versions could embed:

• Up-to-date guideline retrieval

• Safety alerts

• Consensus statements from endocrine/obesity societies.

4. Need for patient-facing validation

• Current study evaluated accuracy through clinicians; future work should:

• Include lay users

• Assess readability and trust

• Examine whether responses alter intentions, adherence, or misconceptions.

5. Broader role of LLMs in chronic disease education

• GLP-1RA therapy is a test case.

• Similar comparative frameworks could be applied to:

• Insulin titration

• Hypertension management

• Statin side-effect queries

• This underscores the scalability of your approach.

⸻

✅ If you want very short “bullet-only” versions for direct insertion:

Introduction short bullets to expand:

• Rising volume of GLP-1RA misinformation across digital and social media channels.

• Increasing reliance on online platforms for obesity-related health information.

• Growing use of LLMs as first-line health information sources, yet limited evaluation in pharmacotherapy contexts.

• Lack of direct comparisons between LLM and internet responses for GLP-1RA therapy.

Discussion short bullets to expand:

• Findings align with broader LLM literature: strong consistency, weaker on emerging evidence.

• Clinical implications of inconsistent internet content and potential patient harm.

• Future LLM optimisation through real-time retrieval and model updating.

• Need for layperson-oriented evaluation and readability testing.

• Potential applicability of this comparative framework to other chronic disease therapies.

If you’d like, I can expand these into full polished paragraphs or insert them into your manuscript with tracked changes.
