# Supplementary material for: Accuracy of Large Language Model Responses Versus Internet Searches for Common Questions About Glucagon-Like Peptide-1 Receptor Agonist Therapy: Exploratory Simulation Study
Source: JMIR Form Res. 2025 Nov 24;9:e78289. doi: 10.2196/78289 (PMC12643393; doi:10.2196/78289)
Supplement: Checklist 1 [file formative-v9-e78289-s004.docx]

**The CHART Checklist**

| HEADING | # | CHART CHECKLIST ITEM | Page #* |
| --- | --- | --- | --- |
| Title & Abstract |  |  |  |
| Title | **1a** | State that the study is assessing one or more generative AI-driven chatbots for clinical evidence or health advice. | 1 |
| Abstract/Summary | **1b** | Apply a structured format, if applicable. | 2-3 |
| Introduction |  |  |  |
| Background | **2a** | State the scientific background, rationale, and healthcare context for evaluating the generative AI-driven chatbot(s), referencing relevant literature when applicable. | 4 |
|  | **2b** | State the aims and research questions including the target audience, intervention, comparator(s), and outcome(s). | 4 |
| Methods |  |  |  |
| Model Identifiers | **3a** | State the name and version identifier(s) of the generative AI model(s) and chatbot(s) under evaluation, as well as their date of release or last update. | 6 |
|  | **3b** | State whether the generative AI model(s) and chatbot(s) are open-source or closed-source/proprietary. | 6 |
| Model Details | **4a** | State whether the generative AI model was a base model or a novel base model, tuned model, or fine-tuned model. | 6 |
|  | **4b** | If a base model is used, cite its development in sufficient detail to identify the model. | 6 |
|  | **4c** | If a novel base model, tuned model, or fine-tuned model is used, describe the pre- and/or post-implementation/deployment data and parameters. | 6 |
| Prompt Engineering | **5a** | Describe the evolution of study prompt development. | 6-7 |
|  | **5ai** | Describe the sources of prompts. | 6-7 |
|  | **5aii** | State the number and characteristics of the individual(s) involved in prompt engineering. | 7 |
|  | **5aiii** | Provide details of any patient and public involvement during prompt engineering. | NA |
|  | **5b** | Provide study prompts. | Supp |
| Query Strategy | **6a** | State route of access to generative AI model. | 6 |
|  | **6b** | State the date(s) and location(s) of queries for the generative AI-driven chatbot(s) including the day, month, and year as well as city and country. | 6 |
|  | **6c** | Describe whether prompts were input into separate chat session(s). | 7 |
|  | **6d** | Provide all generative AI-driven chatbot output/responses | Supp |
| Performance Evaluation | **7a** | Define the ground truth or reference standard used to define successful generative AI-driven chatbot performance. | 7, Table 2 |
|  | **7b** | Describe the process undertaken for generative AI-driven chatbot performance evaluation. | 7 |
|  | **7bi** | State the number and characteristics of team members involved in performance evaluation. | 7 |
|  | **7bii** | Provide details of any patients and public involvement during the evaluation process. | NA |
|  | **7biii** | State whether evaluators were masked to the identity of the generative AI-driven chatbot(s) under assessment. | 7 |
| Sample Size | **8** | Report how the sample size was determined. | 6 |
| Data Analysis | **9a** | Describe statistical analysis methods, including any evaluation of reproducibility of generative AI-driven chatbot responses. | 8 |
|  | **9ai** | Report the measures used for performance evaluation. | 7, Table 2 |
| Results |  |  |  |
|  | **10a** | Report the alignment between generative AI-driven chatbot output and ground truth or reference standard using quantitative or mixed methods approaches as applicable. | 9-10, Table 4 |
|  | **10b** | For responses deviating from the ground truth or reference standard, state the nature of the difference(s). | 9-10, Table 4 |
|  | **10c** | Report the assessment for potentially harmful, biased, or misleading responses. | 9-10 |
| Discussion |  |  |  |
|  | **11a** | Interpret study findings in the context of relevant evidence. | 13 |
|  | **11b** | Describe the strengths and limitations of the study. | 13-14 |
|  | **11c** | Describe the potential implications for practice, education, policy, regulation, and research. | 14 |
| Open Science |  |  |  |
| Disclosures | **12a** | Report any relevant conflicts of interest for all authors. | 15 |
| Funding | **12b** | Report sources of funding and their role in the conduct and reporting of the study. | 15 |
| Ethics | **12c** | Describe the process undertaken for ethical approval. | 8 |
|  | **12ci** | Describe the measures taken to safeguard data privacy of patient health information, as applicable. | 8 |
|  | **12cii** | State whether permission/licensing was obtained for the use of original, copyrighted data. | 8 |
| Protocol | **12d** | Provide a study protocol. | 8 |
| Data availability | **12e** | State where study data, code repository, and model parameters can be accessed. | 15 |

*If in supplementary appendix, indicate “supp” and appendix #, if applicable.
